# Fabrication of Bio-Composite of Piezoelectric/Myrrh Nanofiber Scaffolds for Wound Healing via Portable Gyrospun

**DOI:** 10.3390/pharmaceutics17060717

**Published:** 2025-05-29

**Authors:** Enfal Eser Alenezi, Amalina Amir, Hussain Ali Alenezi, Timucin Ugurlu

**Affiliations:** 1 Department of Pharmaceutical Technology, Faculty of Pharmacy, Marmara University, Başıbüyük 34854, İstanbul, Türkiye; enfaleser@marun.edu.tr; 2 School of Mechanical Engineering, College of Engineering, University Technology MARA, Shah Alam 40450, Malaysia; amalina.amir@uitm.edu.my; 3 Manufacturing Engineering Technology Department, College of Technological Studies, PAAET, Shuwaikh 70654, Kuwait

**Keywords:** bio-composite, piezoelectric, PVDF-TrFE, myrrh, plant extract, nanofiber, wound healing, gyrospun

## Abstract

**Background/Objectives**: Polymeric monoaxial nanofibers are gaining prominence due to their numerous applications, particularly in functional scenarios such as wound management. The study successfully developed and built a special-purpose vessel and device for fabricating polymeric nanofibers. Fabrication of composite scaffolds from piezoelectric poly(vinylidenefluoride-trifluoroethylene) copolymer (PVDF-TrFE) nanofibers encapsulated with myrrh extract was investigated. **Methods**: The gyrospun nanofibers were characterized using SEM, EDX, FTIR, XRD, and TGA to assess the properties of the composite materials. The study also investigated the release profile of myrrh extract from the nanofibers, demonstrating its potential for sustained drug delivery. The composite’s antimicrobial properties were evaluated using the disc diffusion method against various pathogenic microbes, showcasing their effectiveness. **Results**: It was found that an 18% (*w*/*v*) PVDF-TrFE concentration produces the best fiber mats compared to 20% and 25%, resulting in an average fiber diameter of 411 nm. Myrrh extract was added in varying amounts (10%, 15%, and 20%), with the best average fiber diameter identified at 10%, measuring 436 nm. The results indicated that the composite nanofibers were uniform, bead-free, and aligned without myrrh. The study observed a cumulative release of 79.66% myrrh over 72 h. The release profile showed an initial burst release of 46.85% within the first six hours, followed by a sustained release phase. Encapsulation efficiency was 89.8%, with a drug loading efficiency of 30%. Antibacterial activity peaked at 20% myrrh extract. *S. mutans* was the most sensitive pathogen to myrrh extract. **Conclusions**: Due to the piezoelectric effect of PVDF-TrFE and the significant antibacterial activity of myrrh, the prepared biohybrid nanofibers will open new avenues toward tissue engineering and wound healing applications.

## 1. Introduction

Wound management has been one of the most fascinating areas of technological expansion. Wound healing is an intricate tissue regeneration in which the skin reacts to the disruption of anatomical integrity, lost due to various defects or traumas [1,2]. This mechanism occurs through interactions between the extracellular matrix (ECM) and cells, as well as with growth factors [3]. Furthermore, wounds that may arise from various causes can be classified as acute or chronic based on their type and size [4]. The formation of superficial thickness skin defects is considered an acute wound and can be renewed by cell growth [5]. Moreover, chronic wounds require more extensive hospital supervision and expensive wound-healing products. From an economic standpoint, the cost of wound treatment, which commands millions of dollars annually around the globe, is roughly $7000–$10,000 per person in Europe and covers 2–4% of European healthcare funding [6]. Also, patients with diabetes are more likely to have chronic wound infections; in addition, it is expected that the number of diabetic patients will grow to 600 million by 2035. Additionally, till 2026, the wound healing products market is expected to reach an annual economic value of approximately 22 billion [7].

To expedite wound healing procedures, dressings such as creams, gels, natural and synthetic autografts, and allografts are primarily administered to the affected wound region in the clinical setting to accelerate wound healing [8]. Although autografts are the best scenario for patients’ wound treatments, they are not feasible for large wound regions, and they do not show full functionality when a large body surface area is involved [9,10]. Consequently, a therapeutic need exists to develop novel, cost-effective therapy techniques comparable to the ECM to promote wound healing [11]. In this context, submicron and nanoscale polymeric fibers are considered excellent candidates for creating skin-tissue scaffold materials due to their significant advantages, including a high surface-to-volume ratio, porousness, hydrophilic and hydrophobic characteristics, controlled biodegradability, acceptable biocompatibility, and distinctive topographical features that closely mimic the diameter range of fifty to five hundred nanometers of collagen fibrils found in the ECM of biological tissues [12,13,14]. These traits make the fibers vital carriers for bioactive compounds that can be utilized to construct complex drug delivery systems [15].

Various techniques, such as phase separation, melt-blowing, drawing, electrospinning, force spinning, and template synthesis, can generate microscale and nanoscale fibers [16]. On the other hand, these techniques have several downsides, such as low production yields and excessive energy usage [17]. For this reason, the creation of wound dressing patches needs an effective polymeric fiber fabrication process capable of producing an appropriate production rate, cutting down on the amount of time required for manufacturing, and enabling control over the diameter of the fibers and the distribution of their diameters. The proposed research will present this approach.

Piezoelectric materials are esteemed in biotechnology and electronics for their exceptional properties. Poly(vinylidene fluoride-co-trifluoroethylene) (PVDF-TrFE) is a copolymer known for its excellent piezoelectric, pyroelectric, and ferroelectric properties, making it highly suitable for nanofiber applications [18,19]. Recent studies have focused on fabricating PVDF-TrFE nanofibers using electrospinning techniques, which enhance the material’s β-phase content, which is crucial for its piezoelectric response [20]. The development of PVDF-TrFE nanofibers has also been explored for biomedical applications. For instance, the use of PVDF-TrFE in wound healing has been investigated due to its ability to generate surface charges that stimulate cell proliferation and differentiation [21,22,23]. Studies have demonstrated that PVDF-TrFE nanofibers can create biohybrid mats with enhanced antibacterial properties [24,25]. The presented paper shows, for the first time, how to fabricate PVDF-TrFE nanofibres with myrrh extract using a special-purpose gyrospun device in a short processing time.

Recently, more emphasis has been placed on investigating natural substances’ antibacterial characteristics [26]. Plants, vegetables, fruits, and other natural resources may contain natural compounds that are abundant in various bioactive compounds, including alkaloids and flavonoids [27]. With their antibacterial, anti-inflammatory, and antioxidant properties, these elements have a wide range of biological effects. Researchers can use their biological impact in medicine by incorporating these materials into medicinal fibers [28]. Myrrh is a notable medicinal herb employed to address multiple ailments in antiquity, particularly in North Africa, India, and the Arabian territories [29,30,31]. This organism is categorized within the genus Commiphora and the family Burseraceae, characterized by its volatile oil composition, alcohol-soluble resins, and water-soluble gum [32,33]. Myrrh extract is incorporated in several forms, such as nanoparticles [34], oil [35], film, hydrocolloid dressing [36], and, in very limited studies, in the form of polymeric fibers [37]. Various in vitro and in vivo investigations have shown that myrrh and its associated compounds have antibacterial characteristics and possess cytotoxic, antioxidant, antiviral, and antifungal effects [38,39,40,41,42]. It should be noted that the number of investigations that have used polymeric fiber manufacturing methods to fabricate myrrh nanofibers is still minimal [37]. Furthermore, to date, no studies have yet been conducted to investigate the integration of myrrh extract into the PVDF-TrFE nanofiber structure.

This research will investigate the application of nanotechnology and pharmaceutical technology in fabricating wound dressing patches for skin injuries that cannot be treated with conventional methods. From a nanotechnology perspective, novel monoaxial gyrospinning will be utilized to fabricate fibrous materials in the submicron and nanoscale ranges using composite materials, achieving a high production yield in a short timeframe. In addition, encapsulating the natural Myrrh plant, which has antibacterial and anti-inflammatory substances, with (PVDF-TrFE) gyrospun fibers will shed light on pharmaceutical technology. The gyrospun nanofiber scaffolds used as wound dressings aim to accelerate the wound-healing process by enhancing the wound-healing mechanism and addressing the issue of antibiotic resistance. Moreover, the optimal delivery of the encapsulated extract, together with this new pharmaceutical form developed with nanotechnology, will bring a whole new perspective to the treatment of wound areas.

## 2. Materials and Methods

### 2.1. Materials

In this investigation, PVDF-TrFE, (Mw = 450 kg·mol^−1^), a piezoelectric co-polymer powder resin with a composition of 55/45 (mol) and a Curie temperature of 66 °C, was produced by Arkema Piezotech (La Défense, France) and purchased from PolyK Technologies (State College, PA, USA). The myrrh resin was obtained from Esmail Alattar for Natural Herbs (Rawda, Kuwait). Organic solvents, such as dimethylformamide (DMF) (C_3_H_7_NO), ethanol (99.9%, *v*/*v*), and acetone, were purchased from Fisher Scientific (Shah Alam, Malaysia). All materials were used without further treatment or purification.

### 2.2. Fabrication of Myrrh/PVDF-TrFE Nanofibers

#### 2.2.1. Extraction of Myrrh

**Figure 1:**
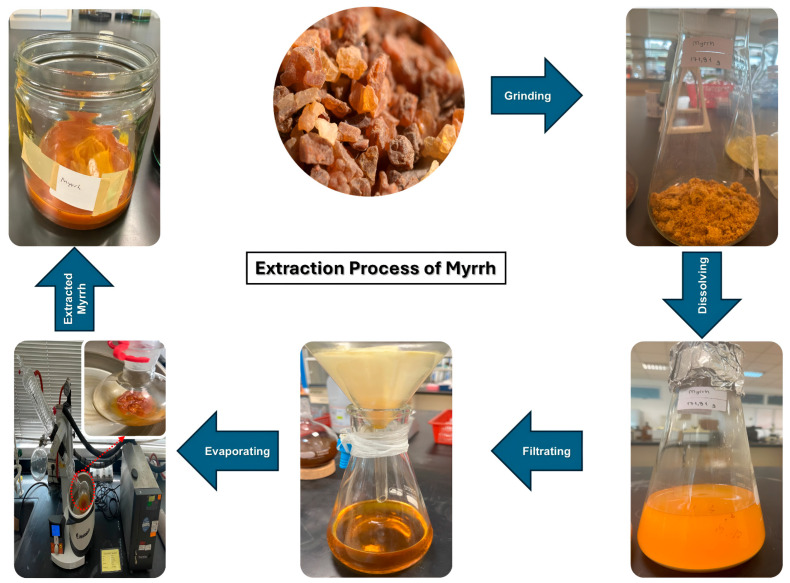
The extraction process of myrrh.

The myrrh resin was washed and cleaned with distilled water, and then it was allowed to air dry before being crushed into a powder (Figure 1). 172 g of powdered myrrh was macerated for 24 h in 1000 cc of ethanol [43]. The gummy extract was obtained by evaporating the solvent using a Heidolph rotary evaporator (Hei-vap Precision model, Schwabach, Germany) at 40 °C for 3 h [44]. After that, it was filtered under a vacuum pump (MZ2CNT model, Wertheim, Germany) using Whatman filter paper number 1. Until use, the resultant extract was stored at −4 °C.

The extraction yield was calculated using the following Equation (1), and all calculations were triplicated:

Yield % = (W1/W2) × 100 (1)

where W1 is the weight of the resulting extract, and W2 is the weight of the myrrh used for the extraction process. The extraction yield for myrrh was 21.36% *w*/*w*.

#### 2.2.2. Preparation of the Myrrh, PVDF-TrFE, and Myrrh/ PVDF-TrFE Solutions

Solutions of PVDF-TrFE at three concentrations, as follows: 18, 20, and 25% *w*/*v*, were prepared by dissolving PVDF-TrFE (5.4, 6, and 7.5 g, respectively) in a mixture solvent of dimethylformamide (DMF) and acetone (18 mL:12 mL) [45,46,47,48], stirring for 6 h and kept at ambient/room temperature (25 °C). For preparing the Myrrh/PVDF-TrFE solution, 400 mg of gummy myrrh extract was dissolved in 10 mL ethanol [49]. Volumes of 1, 1.5, and 2 mL of myrrh extract were added to the PVDF-TrFE solution separately, stirring for four hours, to form a homogeneous solution with a light brown color. Myrrh/ PVDF-TrFE solutions were coded as 0%-Myrrh/ PVDF-TrFE, 10%-Myrrh/ PVDF-TrFE, 15%-Myrrh/ PVDF-TrFE, and 20%-Myrrh/PVDF-TrFE according to the ratio of Myrrh extract with respect to PVDF-TrFE (v) as 0.0, 10, 15, and 20% *v*/*v*, respectively. All the measurements were repeated three times at ambient temperature.

#### 2.2.3. Fabrication of the Scaffolds by Gyrospining

**Figure 2:**
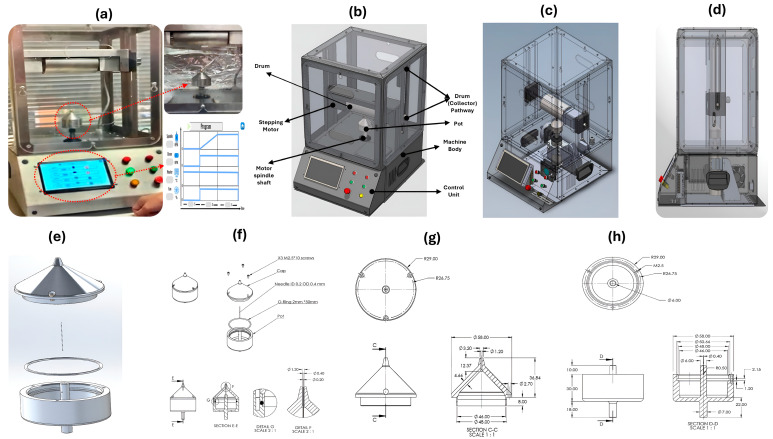
The parts of the gyrospun (**a**) full view of constructed gyrospun, (**b**) designed model with device parts, (**c**) transparent view, (**d**) device side-view, (**e**) pot disassemble, (**f**) pot main parts, (**g**) pot cap dimensions, and (**h**) pot detailed dimensions.

Figure 2a,b illustrate the proposed portable monoaxial device designed and constructed for this investigation. As shown in Figure 2c, the device consists of the following main parts: the device body, with dimensions of 374 × 466 × 604 mm; the drum (collector) holder, with dimensions of 323 × 82 × 83 mm, which holds the collector drum with a diameter of 76 mm and a length of 144 mm. Moreover, the drum can be allocated and is adjustable to move from 0 to 225 mm from the pot orifice via a vertical pathway, as illustrated in Figure 2d. In addition, as shown in Figure 2e, the crucial part of the device is the pot or polymer solution chamber, which consists of a base with a shaft that allows the pot to be connected to the motor spindle, which spins up to 10 k revolutions per minute and the pot cap, as illustrated in Figure 2f, showing a disassembled view and all dimensions of the pot represented in Figure 2g,h.

The manufacturing method for nanofibers is quite delicate, and even a slight variation of milliseconds can significantly impact the result. All regulated and monitored parameters, such as spinning speed, drum (collector) speed, and cabinet temperature, should be overseen by the integrated controllers’ ICs. The machine’s foundation is an Arduino Nano, with 22 input/output pins and a clock speed of 16 MHz. Using an HMI, as shown in Figure 2a, the Arduino can gather user instructions; this configuration eliminates the need for traditional control switches and timers, saving much time when setting up and changing control settings. In addition to the touchscreen, the device features standard start, stop, and emergency buttons. All these switches are part of a latch circuit.

The experiments were conducted by pouring 2 mL of the polymer solution into the pot and spinning it at the optimum speed, which was determined to be 8000 rpm. The collector distance was 160 mm, and the drum speed was 100 rpm. Moreover, the optimum relative humidity (RH%) for fabricating nanofibers was 45–49%.

### 2.3. Characterization of Composite Fibers

#### 2.3.1. Scanning Electron Microscopy (SEM)

The size and morphology of the nanofibers were examined using scanning electron microscopy (SU3500, Hitachi, Tokyo, Japan). Before imaging, the samples’ surfaces were coated with gold (Au) for approximately 60 s using a sputter coating machine (JFC-1600, JEOL Tokyo, Japan). The applied accelerating voltage was 15 kV, and the working distance was 10 mm. The average fiber diameter and size distribution were determined by measuring 100 fibers in randomly recorded SEM micrographs using ImageJ version 1.54p (LOCI, University of Wisconsin, Madison, WI, USA).

#### 2.3.2. Energy-Dispersive X-Ray Spectroscopy (EDX)

The elemental analysis was used to assess the energy and intensity distribution of the produced P(VDF-TrFE and P(VDF-TrFE)/Myrrh nanofibers, analyzed by combined SEM/EDX (SU3500, Hitachi, Tokyo, Japan) mapping techniques.

#### 2.3.3. Fourier Transform Infrared Spectroscopy (FTIR)

FTIR measurements were conducted using a PerkinElmer Spectrum One spectrometer (East Lyme, CT, USA), and the resulting spectrograms were analyzed with OPUS Viewer version 6.5 software to assess the molecular composition of the fibrous patches and verify the presence of myrrh within these patches. The analysis was performed at room temperature (24 °C) in transmission mode, specifically within the range of 4000 cm^−1^ to 515 cm^−1^. The experiments utilized 32 scans at a resolution of 4 cm^−1^. The spectra were analyzed using OPUS Viewer version 6.5 (Billerica, MA, USA).

#### 2.3.4. X-Ray Powder Diffraction (XRD)

XRD was performed using a D/Max-BR diffractometer (RigaKu, Tokyo, Japan) to examine the fibers’ crystalline form and structure. Analyses were conducted at 40 kV and 40 mA over a 2θ range of 5–90° at a 2°/min rate. OriginPro 2024.b software (OriginLab Corporation, Northampton, MA, USA) was used to convert the obtained data into diffractograms.

#### 2.3.5. Thermogravimetric Analysis (TGA)

Thermogravimetric sample analysis evaluated the Myrrh/PVDF-TrFE nanofiber thermal stability by measuring its weight change relative to temperature and time. A Pyris STA 6000 simultaneous thermal analyzer (PerkinElmer, Springfield, IL, USA) was utilized. Approximately 5 mg of Myrrh/PVDF-TrFE nanofiber was placed on a sample pan, and the balance device measured the weight before and after heating. The temperature range was set from 20 to 500 °C with a heating rate of 10 °C/min, and the sample was heated in a controlled environment using nitrogen gas at a flow rate of 70 mL/min. Measurements were performed in duplicates.

#### 2.3.6. Encapsulation Efficiency

Encapsulation efficiency (EE) is defined as the weight ratio of the incorporated drug within the gyrospun nanofiber patches to the initial quantity of plant extract utilized in the formulation. The following standard test method determines the myrrh content in the gyrospun nanofibers. The nanofibers dissolved entirely in the solvent, with UV detection occurring at 415 nm for myrrh [49]. After weighing 5 mg, PVDF-TrFE/Myrrh nanofibers were dissolved in 10 mL of Myrrh solvent in a volumetric flask. The flask was swirled gently for 4 h to ensure the complete dissolution of myrrh from the nanofibrous scaffolds into the solvent. Three milliliters of solution were obtained and analyzed using a UV-Vis/NIR spectrophotometer at 415 nm (The JASCO UV-Vis/NIR Spectrophotometer V-670, Tokyo, Japan). The percentage encapsulation efficiency and drug loading in patches were determined using Equations (2) and (3), PVDF-TrFE gyrospun fibrous meshes for the controlled release of myrrh.

DL (%) = 100 ×(Weight of myrrh entrapped in the sample/weight of the patch) (2)

EE (%) = 100 ×(Determined drug content/theoretical drug content)(3)

#### 2.3.7. In Vitro Drug Release

The JASCO UV-Vis/NIR Spectrophotometer V-670 Series was utilized for loading and release investigations. Since the myrrh extract consists of many compounds, the authors conducted their research to obtain the bioactive compound of the myrrh extract, named total flavonoids (TF), at a wavelength of 415 nm corresponding to the absorbance peak of the TF content, utilizing a UV-Vis spectrophotometer [49]. The TF in myrrh is chosen for its antioxidant properties, which help neutralize free radicals, reduce oxidative stress, and protect cells from damage. Also, TF has antimicrobial activity, contributing to myrrh’s ability to fight against various pathogens, including bacteria and fungi. In addition, total flavonoids also have anti-inflammatory effects and can reduce inflammation, which is beneficial for conditions like arthritis and other inflammatory diseases [50]. One milligram of each fiber sample was placed into microtubes, to which three milliliters of PBS were added. Each sample was continuously agitated with mild shaking in the shaker throughout the experiment. Each sample duration was 48 h, during which 1 mL of eluent (solvent) was extracted at intervals of 30 min, 1, 2, 4, 6, 8, 12, 24, 36, 48, and 72 h, and subsequently replaced with 1 mL of fresh PBS (pH 7.4 at 37 °C). One milliliter of the eluent was transferred to cuvettes for injection into the UV-Vis spectrophotometer at a wavelength of 415 nm. Absorbance values were measured at the designated time intervals for each fiber sample. A calibration curve was constructed based on the absorbance values of myrrh.

#### 2.3.8. Antimicrobial Activity

##### Disk Diffusion Approach for Antimicrobial Evaluation

Five applied pathogens were used, including two Gram-positive bacteria, *Staphylococcus aureus* ATCC 25,923 (*S. aureus*) and *Streptococcus mutans* ATCC 25,175 (*S. mutans*), and two Gram-negative bacteria: *Escherichia coli* ATCC 25,922 (*E. coli*) and *Salmonella typhimurium* ATCC 14,028 (*S. typhimurium*), with *Candida albicans* ATCC 10,231 (*C. albicans*) representing the unicellular fungi. The pathogens’ preculture was prepared overnight by incubating each pathogen under shaking conditions (150 rpm) in the nutrient broth at 37 °C. The antimicrobial activity of the nanofibres PVDF-TrFE loaded with 20, 15, and 10% myrrh extract was assessed using the disk diffusion method. Specifically, 100 µL of serially diluted pathogenic cultures were uniformly inoculated onto Mueller-Hinton agar plates. Disks (3 mm in diameter) prepared from the PVDF-TrFE/myrrh nanofibres composite membrane, functionalized with different extract concentrations, were placed on the agar surface. Before testing, all membranes were sterilized under ultraviolet (UV) light for 30 min to ensure sterility. The plates were stored at 4 °C for 2 h to allow complete diffusion of the myrrh extract. Following this, the inoculated plates were incubated for 24 h under static conditions at 37 °C. The inhibitory effect of myrrh extract was evaluated by measuring the developed halo-zone diameter, including the agar well.

## 3. Results and Discussion

### 3.1. Morphological Characterization of Fibers

**Figure 3:**
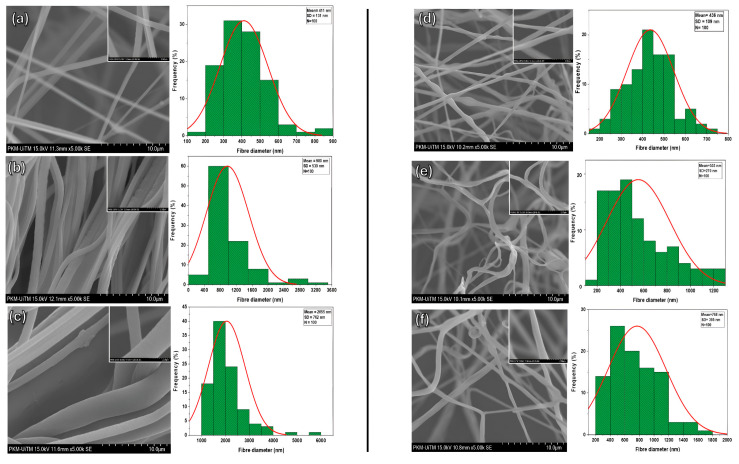
SEM images and fiber diameter distributions of (**a**) 18% PVDF-TrFE, (**b**) 20% PVDF-TrFE, (**c**) 25% PVDF-TrFE, (**d**) PVDF-TrFE/myrrh at 10% (*v*/*v*) myrrh, (**e**) PVDF-TrFE/myrrh at 15% (*v*/*v*) myrrh, and (**f**) PVDF-TrFE/myrrh at 20% (*v*/*v*) myrrh.

Figure 3a–c illustrate the characteristics of fibers fabricated by the gyro spin for virgin PVDF-TrFE at concentrations of 18%, 20%, and 25%, respectively. In the 18% scenario, the mean diameter for the 100 randomly selected fibers was 411 ± 130 nm. In contrast, the maximum and minimum diameters of the 100 fibers measured at this concentration were 885 nm and 119 nm, respectively. When the concentration increased to 20%, the average diameter measured was 980 ± 530 nm, with a maximum diameter of 3272 and 394 nm. This clearly showed that the fibers had a nonhomogeneous distribution and stuck to each other, indicating semi-aligned fibers. Figure 3c illustrates the 25% PVDF-TrFE fibers, which have a mean diameter of 2055 ± 762 nm, with a maximum diameter of 5946 nm and a minimum diameter of 1018 nm. The distribution of fibers in this particular sample, which has dimensions extending beyond the nanoscale, was found to be highly uneven. From the above results, the fibers produced by the proposed gyrospun method had a smaller size and better morphology and distribution at 18% compared to 20% and 25%; therefore, this concentration was selected for use in the PVDF-TrFE/myrrh composite fibers manufacturing study. Figure 3d–f show the morphology of fibers and the homogeneity of fiber distribution of PVDF-TrFE/myrrh at 10%, 15%, and 20% (*v*/*v*) myrrh, respectively. As shown in Figure 3d, the mean fiber diameter was observed to be the smallest at the 10% myrrh sample, with a value of 436 ± 109; the maximum and minimum fiber diameters were 748 and 170 nm, respectively.

In contrast, the average fiber diameter increases from 15% to 553 ± 270 nm, compared to 10%, with maximum and minimum fiber diameters of 1230 and 197 nm, respectively. Figure 3f presents the 20% myrrh, where the mean fiber diameter increased to 768 ± 395 nm, with a maximum diameter value of 1628 nm and a minimum fiber diameter of 266 nm. The above results clearly show that the fiber diameter is directly proportional to the myrrh concentration. As the myrrh content increased, the fiber diameter also increased. This is because the plant extract (myrrh) with a higher concentration, whose viscosity increases, can decelerate the evaporation of the polymer solution solvent and change it from a liquid to a solid phase, which makes it harder for the solution to stretch into finer fibers during processes. For example, in forcespinning, electrospinning, and pressurized gyration techniques, the flow dynamics are influenced by concentration; therefore, a higher concentration can lead to more stable and thicker fiber formation [51,52,53].

### 3.2. Elemental Characterization of Fibers

**Figure 4:**
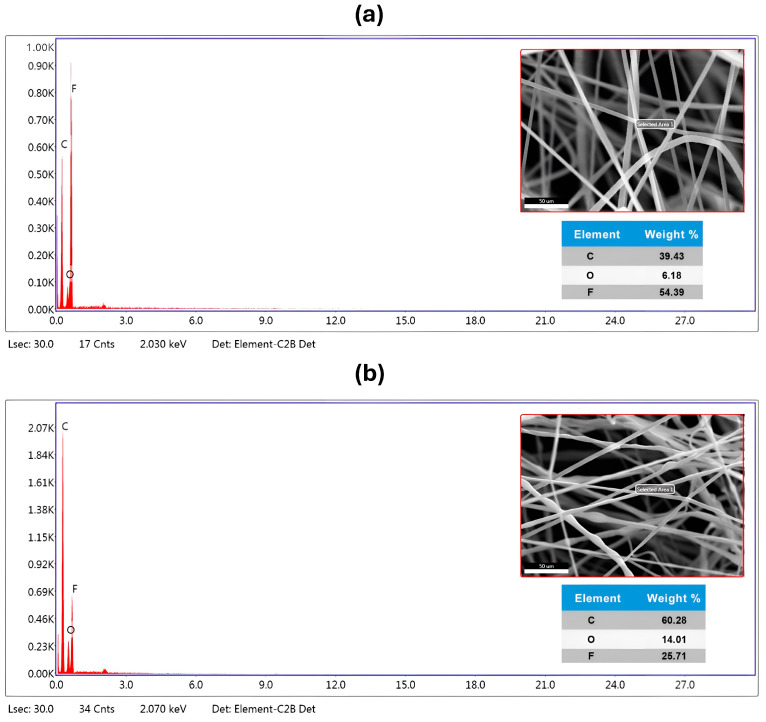
SEM image, elemental dispersive spectrum, and elements composition percentage of (**a**) pure PVDF-TrFE nanofibers, a concentration of 18%, and (**b**) bio-composite PVDF-TrFE encapsulated with 10% (*v*/*v*) Myrrh.

EDX analysis is a method used for the elemental assessment or chemical verification of the manufactured PVDF-TrFE and PVDF-TrFE/Myrrh nanofiber samples [54]. An elemental analysis was performed to determine the concentrations of carbon (C), fluorine (F), and oxygen (O) atoms. The elemental composition and atomic weight % analysis of the produced nanofiber structure is shown in Figure 4.

Furthermore, Figure 4a presents the EDX analysis of pure PVDF-TrFE nanofibers, revealing a robust signal for fluorine at 2.150 keV in conjunction with carbon and oxygen peaks, thereby confirming its uniform distribution and high purity. Furthermore, the F element was identified in the pure PVDF-TrFE nanofiber at 56.31%, whereas O and C were discovered at 27.97% and 39.43%, respectively.

The composite PVDF-TrFE/Myrrh nanofibers (Figure 4b) exhibit a homogeneous distribution. Distinct sharp peaks are observed in the EDX spectra of P(VDF-TrFE)/Myrrh nanofibers, corresponding to carbon (Kα radiation at 0.277 keV) and fluorine (Kα radiation at 0.677 keV). The inclusion of Myrrh extract into PVDF-TrFE) nanofiber resulted in observable alterations in the energy levels of the peaks. While the atomic weight percentages of carbon and oxygen increased, the nuclear weight percentage of fluorine declined markedly.

Ultimately, EDX elemental analysis was used to verify the existence of a composite of PVDF-TrFE with the capsulated myrrh, affirming the presence of identical atoms in the analyzed sample with high purity. The EDX elemental analysis confirms the presence of all constituents in the nanofibers, demonstrating exceptional purity.

### 3.3. Fourier Transform Infrared Spectroscopy (FTIR)

**Figure 5:**
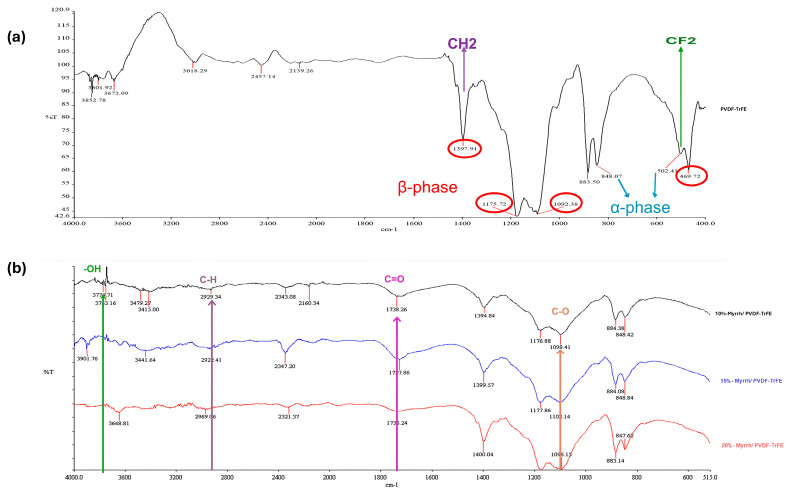
FTIR spectra of (**a**) pure PVDF-TrFE nanofiber (Red circles indicate the β-phase, and blue arrows indicate the α-phase), and (**b**) 10%, 15%, and 20% Myrrh/PVDF-TrFE nanofibers.

FTIR analysis was performed to identify the molecular structures of the Myrrh extract and PVDF-TrFE, as well as the Myrrh-loaded PVDF-TrFE fibrous patches produced by gyro spinning. In all concentrations of myrrh (10, 15, and 20%), bands between 3000 and 3700 cm^−1^ are observed in Figure 5, corresponding to broad peaks attributed to the stretching vibrations of hydroxyl groups in alcohols and phenols, which are characteristic of the myrrh extract. Another characteristic band, assigned to a range of 2920 to 2930 cm^−1^, corresponds to the symmetric and asymmetric stretching of the aliphatic C-H bonds [55]. A sharp and weak peak at 1738 cm^−1^, corresponding to the C=O (carbonyl groups), was observed for 10% Myrrh/PVDF-TrFE NFs, indicating the presence of both ester and carboxyl groups [56]. The absorption peak at around 1050 cm^−1^ corresponds to the C-O stretching [57].

PVDF-TrFE copolymer indicates a characteristic FTIR spectrum. Regarding the abundance of fluorine atoms, considerable infrared-active vibrations for PVDF-TrFE are concentrated in a relatively narrow range, from 400 to 1500 cm^−1^. Getting information regarding the absorption peak assignments in the published literature is possible [58,59,60,61].

The FTIR spectra for PVDF-TrFE NFs and 10%-myrrh/PVDF-TrFE NFs were nearly identical, demonstrating prominent absorption bands related to the alpha-phase at 502 and 848 cm^−1^, and the beta-phase at 469, 883, 1092, 1175, and 1397 cm^−1^. The bands at 502 cm^−1^ and 1397 cm^−1^ correspond to the CF2 bending mode and the CH2 waggling vibration, respectively, whereas the band at 850 cm^−1^ is attributed to the CF2 symmetric stretching mode [62,63].

### 3.4. XRD Analysis of Gyrospun Nanofibers

**Figure 6:**
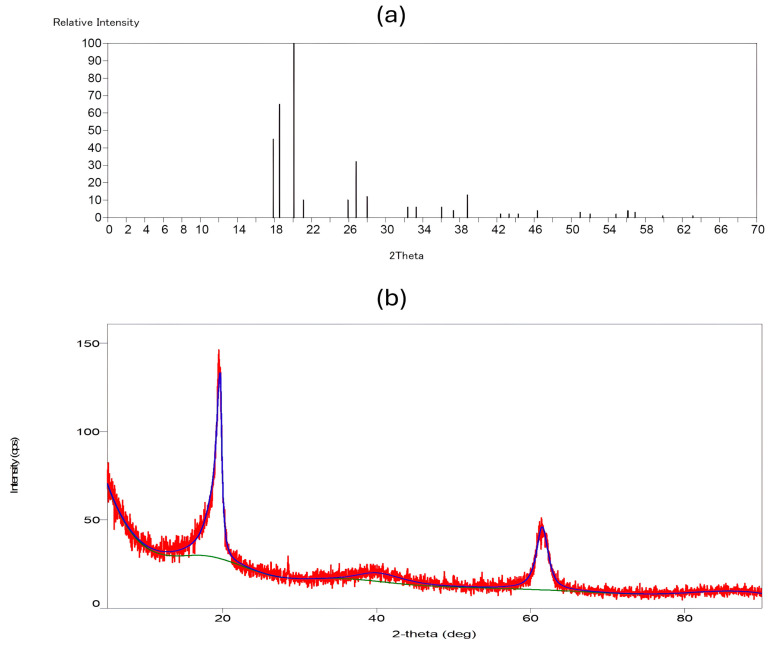
XRD pattern graphs: (**a**) PVDF-TrFE, and (**b**) Myrrh/PVDF-TrFE scaffold. (The red curve in the XRD pattern indicates experimental data obtained during the study of PVDF-TrFE encapsulated with myrrh. The green curve depicts the background signal, which was eliminated from the experimental data to isolate the peaks. The blue curve represents the fitted data, which aids in identifying and quantifying the crystalline phases present.

The XRD technique is an effective method for verifying the inclusion of PVDF-TrFE and Myrrh extract in polymer nanofibers by examining their impact on their crystallinity. Figure 6a illustrates the XRD spectra of PVDF-TrFE nanofiber patch and 10% Myrrh/PVDF-TrFE nanofiber patche in Figure 6b, respectively.

The unadulterated PVDF-TrFE nanofiber exhibited a peak at 20.11°, indicative of the 200/110 reflections of the β phase crystal, which is responsible for piezoelectric capacity. The β phase is detectable by its distinctive peak at approximately 2θ = 20° within the (1 1 0) and (2 0 0) planes with regard to the literature [64,65,66]. This phase was indicated by a peak at 2θ = 20.11°, associated with the (1 1 0/2 0 0) planes of the β-phase crystal structure. The α phase, characterized by peaks at approximately 17.86° and 18.56° corresponding to the (0 2 0) plane, was observed in the PVDF-TrFE nanofibers.

Conversely, for the 10%-Myrrh/PVDF-TrFE NFs, it was observed that the peak width at 20.11° shifted to 19.65°, becoming slightly narrower and more intense following the integration of the extract. Additionally, reflections at 2θ values of 40.21° and 61.42° emerged, which can be attributed to the amorphous phase of myrrh [34].

XRD spectra provided further evidence that our nanofiber patches had the ideal crystalline structure, demonstrating their piezoelectric ability derived from the β phase. The extraordinary piezoelectric properties of PVDF-TrFE are attributed to the β-phase, which is responsible for producing the most substantial dipole moment [67,68]. The spectra also showed that the nanofiber matrix contains myrrh extract, which is naturally crystalline [60].

### 3.5. Thermogravimetric Analysis (TGA)

**Figure 7:**
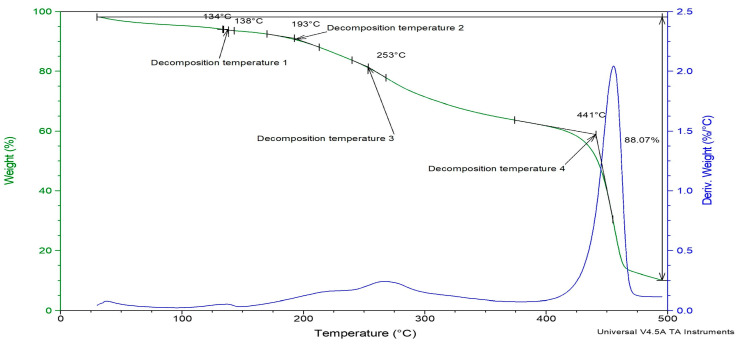
TGA analysis of Myrrh/PVDF-TrFE scaffold.

The influence of PVDF-TrFE and myrrh extract on the thermal stability of nanofibers was examined using TGA analysis. Figure 7 illustrates the thermogram’s weight loss and derivatives as a temperature function for 10%-Myrrh/PVDF-TrFE nanofibers. According to TGA, poly (vinylidene tetrafluoroethylene) (P(VDF-TrFE)) has exceptional thermal stability. Decomposition often starts at 420–430 °C, showing that the material can resist high temperatures before significant degradation. Weight loss is highest between 430 °C and 500 °C, resulting from dehydrochlorination, which releases hydrogen fluoride (HF) and causes polymer backbone scission [69].

The nanofiber patch deteriorated in four stages under heat. The sample exhibited a derivative weight loss of less than 0.25% up to 150 °C, which coincides with the melting point of the PVDF-TrFE polymer.

The initial step reached a peak temperature of 138 °C while the subsequent step attained a peak of 193 °C, resulting in a weight loss of less than 10% of the nanofiber patch up to this point. The third breakdown temperature occurred between 200 °C and 300 °C, peaking at 253 °C, whereas the derivative loss for the nanofiber patches was approximately 0.5% by weight.

The most significant observable alteration occurred between 400 °C and 500 °C, featuring a decomposition peak at 441 °C. When comparing the extract-loaded PVDF-TrFE nanofiber with a pure one, there is a slight increase in temperature, since pure P(VDF-TrFE), which begins to degrade around 420 °C. This shift can be caused by the fact that the drug enhances the thermal stability of the polymer, likely due to intermolecular interactions, increased crystallinity, or barrier effects, since the extract may act as a physical barrier within the PVDF-TRFE matrix [70]. Also, due to the functional groups that myrrh extract contains, it might stabilize the polymer against thermal degradation [71]. Subsequently, the gyrospun nanofiber patches exhibited a 2.25% reduction in derivative weight. Thermogravimetric analysis (TGA) reveals the enhanced thermal stability of the herbal medicine myrrh within the nanofiber patch. Consequently, the polymer safeguards the medication against thermal breakdown.

### 3.6. In Vitro Drug Release

**Figure 8:**
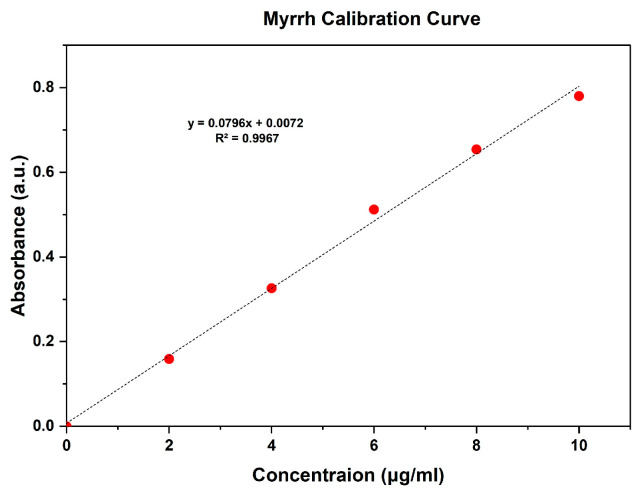
Calibration curve of myrrh.

In vitro drug release assays were conducted using PVDF-TrFE/Myrrh gyrospun nanofiber patch specimens. Firstly, UV spectra were used to determine the concentration range of myrrh, ranging from 2 to 10 μg/mL, which resulted in establishing a linear standard calibration curve based on myrrh absorption values (R² = 0.9967). The equation is y = 0.0796x + 0.0072, where x represents the absorbance and y represents the concentration in μg/mL. This spectrum was utilized to assess medication release (Figure 8) quantitatively.

Monitoring drug release profiles over a prolonged period is essential for enhancing medicine performance, minimizing adverse reactions, and reducing the frequency of drug administration.

Consequently, cumulative drug release assays were conducted over a 72-h period using drug-loaded samples to examine the release profile of myrrh in phosphate-buffered saline (PBS) at pH 7.4 and 37 °C, simulating the physiological conditions of living organisms.

**Figure 9:**
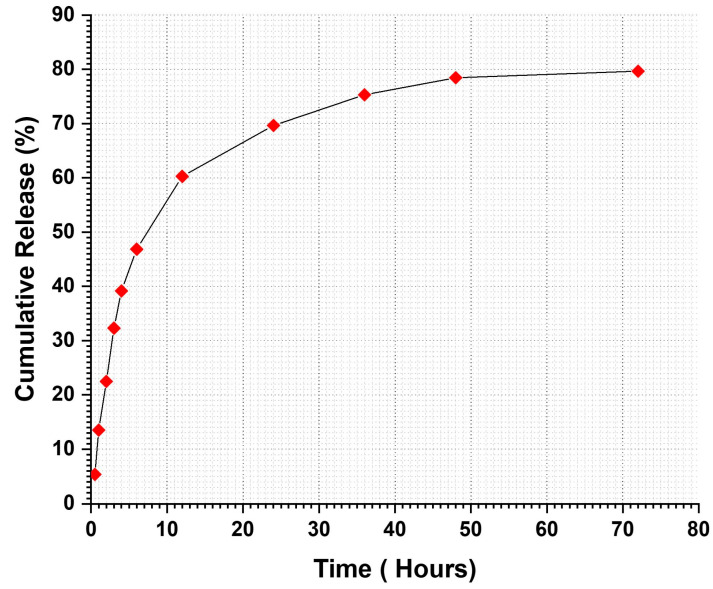
In vitro drug release profile of Myrrh/PVDF-TrFE nanofibrous scaffolds.

The release curve primarily consisted of two phases: an initial “burst release” followed by a “slow release”, which led to the occurrence of the sustained release phase as illustrated in Figure 9. Myrrh was released in a burst manner at the end of 6 h, achieving a 46.85% release, followed by a sustained release of 79.66% over the remainder of the testing time. The initial burst phenomenon is defined by myrrh extract release from the nanofiber, attributed to extract molecules situated on or near the fiber surface that disintegrate swiftly upon contact with the release medium. This release may have transpired during the gyrospinning production process, wherein some of the extract may not be entirely encapsulated within the polymer matrix but rather is superficially dispersed. Moreover, the nanofibers’ high surface area-to-volume ratio and porous architecture facilitate the diffusion of the surface-bound extract into the release medium.

The initial burst release is advantageous for administering antimicrobial medications, as it is crucial to eliminate proliferating bacteria before they begin to replicate [72]. Therefore, continuous administration of antimicrobial agents is necessary to safeguard the residual population of various organisms that endure the first release [73]. Following the burst release phase, an increase in drug release was observed, attributed to the concentration of myrrh in the patches. These rises exhibited a linear and sustained pattern over three days.

Subsequently, encapsulation efficiency (EE) was evaluated to confirm the successful incorporation of myrrh into the nanofibers, yielding a value of 89.8% for 10%-Myrrh/PVDF-TrFE nanofibers. The drug loading efficiency results for the 10% PVDF-TrFE/Myrrh nanofiber patches exhibited 30% drug loading, signifying the effective integration of the drug within the carrier matrix.

### 3.7. Antimicrobial Activity

**Figure 10:**
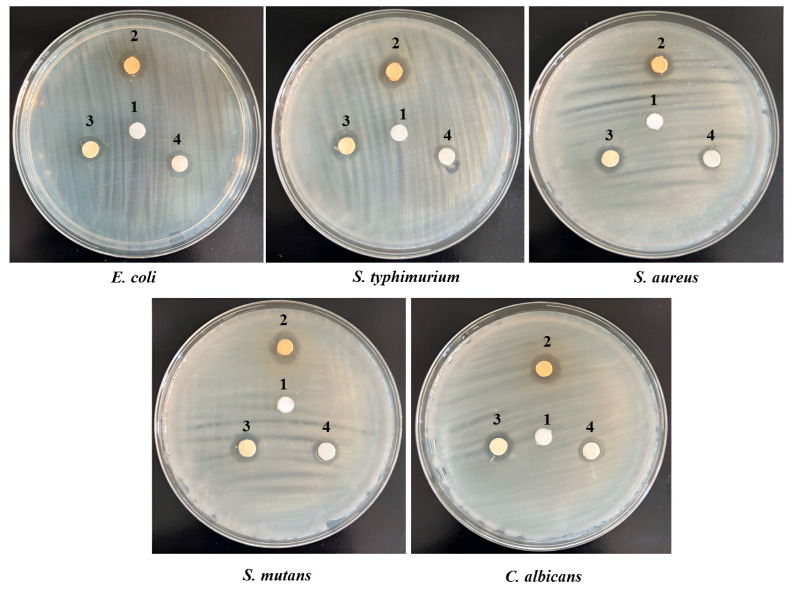
Antimicrobial activity assessments by disk diffusion method for PVDF-TrFE loaded with three concentrations of myrrh extract: 1 (control- PVDF-TrFE free extract), 2 (20% myrrh extract), 3 (15% myrrh extract), and 4 (10% myrrh extract), tested against five pathogenic microbes.

The antimicrobial activity of the developed PVDF-TrFE/myrrh nanofiber patches functionalized with varying concentrations of myrrh extract was tested against five pathogenic microbes, as shown in Table 1 and Figure 10. The antimicrobial activity showed inhibition zones ranging from 7 ± 0.2 to 15 ± 1.5 mm based on the concentration of myrrh extract used. The results revealed that the broad-spectrum antibacterial activity of PVDF-TrFE/myrrh extract was observed at different concentrations against all tested pathogens. Notably, the PVDF-TrFE with a 20% myrrh extract concentration exhibited significantly more vigorous antibacterial activity, followed by 15% and then 10% myrrh extract, indicating that higher myrrh extract levels corresponded to greater antimicrobial effectiveness. In contrast, the PVDF-TrFE without myrrh extract showed no activity, which was used as the negative control against all tested pathogens. The results observed that the *S. mutans* showed high sensitivity against myrrh extract, with no significant differences between other tested pathogens.

## 4. Conclusions

This research successfully designed and constructed a special-purpose mono-axial gyrospun, capable of jetting a polymer solution upstream to fabricate submicron and nanoscale fibers at optimum parameters. Furthermore, as a plant-based therapeutic agent, Commiphora myrrh was successfully extracted. Three different PVDF-TrFE concentrations (18, 20, and 25%, respectively) were spun and fiberized using the proposed gyrospinning method. The lowest mean diameter (411 ± 131 nm) was achieved at 18%, which was selected as the matrix for the myrrh extract. In contrast, the most nonhomogeneous distribution of fibers was obtained at 25%; noteworthy, the highest concentration provides more aligned fibers, but fiber diameter increases markedly. The bio-composite scaffold of PVDF-TrFE and myrrh was successfully fabricated at three different myrrh concentrations (10%, 15%, and 20% *v*/*v*), and a minor mean diameter with homogeneous and fiber distribution was achieved at 10%. FTIR spectra confirmed the interaction between the myrrh extract and PVDF-TrFE via the functional groups. Moreover, XRD has shown that gyrospun PVDF-TrFE nanofibers retain their piezoelectric properties, particularly with an enhanced content of β-phase crystals. The crystallinity and thermal stability of the nanofibers, as confirmed by XRD and TGA, respectively, demonstrate the permanence of the polymer and extract while also revealing that efficiently prepared gyrospun nanofiber scaffolds effectively encapsulate the myrrh. Moreover, the results obtained from the drug release study demonstrate that the sustained release of myrrh from the nanofibers has a high potential for wound treatment. The results demonstrate the potential of nano-sized beadless composite patches, which have novel treatment strategies for transdermal delivery. Functionalization of piezoelectric copolymer PVDF-TrFE incorporating myrrh extract exhibited enhanced antimicrobial efficacy, particularly at higher concentrations, 20%, followed by 15%, and then 10%. Finally, future studies will include comprehensive biocompatibility and in vitro/in vivo functioning investigations of the device, as well as an analysis of the bioactivity of the released extract.

## Figures and Tables

**Table 1:** The antimicrobial activity of PVDF-TrFE loaded with three different concentrations of myrrh extract, as evaluated through the disk-diffusion approach.

Organism	Inhibition Zone Diameter (mm)			
	Polymer	PVDF-TrFE/Myrrh Extract (10%)	PVDF-TrFE/Myrrh Extract (15%)	PVDF-TrFE/Myrrh Extract (20%)
*E. coli*	0.0 ± 0.0	8 ± 0.8	11 ± 1.2	12 ± 1.8
*S. typhimurium*	0.0 ± 0.0	7 ± 0.4	11 ± 1.5	13 ± 1.5
*S. aureus*	0.0 ± 0.0	7 ± 0.2	9 ± 1.04	11 ± 1.3
*S. mutans*	0.0 ± 0.0	9 ± 1.05	12 ± 1.7	15 ± 1.5
*C. albicans*	0.0 ± 0.0	8 ± 0.2	10 ± 1.3	12 ± 1.7

## Data Availability

The data presented in this study are available on request from the corresponding author.
